# Incidence of TB and HIV in Prospectively Followed Household Contacts of TB Index Patients in South Africa

**DOI:** 10.1371/journal.pone.0095372

**Published:** 2014-04-23

**Authors:** Cari van Schalkwyk, Ebrahim Variava, Adrienne E. Shapiro, Modiehi Rakgokong, Katlego Masonoke, Limakatso Lebina, Alex Welte, Neil Martinson

**Affiliations:** 1 The South African Department of Science and Technology/National Research Foundation (DST/NRF) Centre of Excellence in Epidemiological Modelling and Analysis (SACEMA), University of Stellenbosch, Stellenbosch, South Africa; 2 Klerksdorp/Tshepong Hospital Complex, North West Department of Health, Klerksdorp, South Africa; 3 University of the Witwatersrand, Johannesburg, South Africa; 4 University of California San Francisco, Department of Internal Medicine, San Francisco, California, United States of America; 5 Perinatal HIV Research Unit, University of the Witwatersrand, Johannesburg, South Africa; 6 Center for TB Research, Johns Hopkins University School of Medicine, Baltimore, Maryland, United States of America; Institute of Infectious Diseases and Molecular Medicine, South Africa

## Abstract

**Objective:**

To report the incidence rates of TB and HIV in household contacts of index patients diagnosed with TB.

**Design:**

A prospective cohort study in the Matlosana sub-district of North West Province, South Africa.

**Methods:**

Contacts of index TB patients received TB and HIV testing after counseling at their first household visit and were then followed up a year later, in 2010. TB or HIV diagnoses that occurred during the period were determined.

**Results:**

For 2,377 household contacts, the overall observed TB incidence rate was 1.3 per 100 person years (95% CI 0.9–1.9/100py) and TB incidence for individuals who were HIV-infected and HIV seronegative at baseline was 5.4/100py (95% CI 2.9–9.0/100py) and 0.7/100py (95% CI 0.3–1.4/100py), respectively. The overall HIV incidence rate was 2.2/100py (95% CI 1.3–8.4/100py).

**Conclusions:**

In the year following a household case finding visit when household contacts were tested for TB and HIV, the incidence rate of both active TB and HIV infection was found to be extremely high. Clearly, implementing proven strategies to prevent HIV acquisition and preventing TB transmission and progression to disease remains a priority in settings such as South Africa.

## Background

South Africa has an estimated annual tuberculosis (TB) incidence rate of 1.0 per 100 population (95% CI 0.8–1.2) [1]. This is largely fuelled by a severe and generalised human immunodeficiency virus (HIV) epidemic, which continues to be propagated by HIV incidence of 1.3 per 100 susceptible individuals per annum in 15–49 year olds [2]. There are several studies from high burden countries describing the prevalence of HIV and TB in contacts of TB index cases, but few describe TB incidence over a prospective follow up period, and none, that we are aware of, where HIV incidence was determined simultaneously [3], [4], [5], [6]. Incidence measures of both TB and HIV are important for those planning or implementing household-based active case finding strategies, which have shown some promise in controlling TB [7], [8], and for policy makers to base decisions on the utility of a second or third visit to the household of an index TB patient. They are also critical in estimating sample sizes for clinical trials of preventive treatment in household contacts. This study describes the incidence of TB and HIV in household contacts of TB index patients in a very high TB and HIV burden setting.

## Methods

### Ethics Statement

This study was approved by the ethics committees of the University of the Witwatersrand, the Research Committee of the Klerksdorp/Tshepong Hospital Complex, and the Johns Hopkins School of Medicine institutional review board. All study participants gave individual written informed consent for study participation. Written parental consent was obtained for all participants younger than 18, with assent from 7–17 year olds. Separate written consent for on-site rapid HIV testing or oral specimen collection for HIV testing was obtained.

### Study setting

This prospective cohort study was conducted in the households of index TB patients in the Matlosana sub-district in North West Province. TB patients were recruited from the adult internal medicine wards of the only public sector regional hospital serving the sub-district, and from the 16 primary care clinics within the sub-district. The entire district within which Matlosana is situated, is estimated to have an annual TB incidence of close to 1.2 per 100 person years (personal communication L. Mvusi) and HIV seroprevalence of 29% [9] in pregnant women. The HIV seroprevalence in South Africa among all persons aged 15–49 was 17.9% in 2012 [10].

### Baseline study visit

We recruited index TB patients into a study that previously reported the prevalence of undiagnosed TB and HIV in contacts of adult TB index cases recruited at a regional hospital and its feeder clinics [11]. Patients were eligible if they were admitted to hospital with a diagnosis of TB, irrespective of the presence of laboratory confirmation but, if recruited from a community clinic, they were required to be sputum smear positive.

Households had a baseline first visit between February and November 2009. At that visit, individually consenting household members were offered on-site rapid HIV testing after counselling; those who were found to be HIV-infected had post-test counselling and a blood draw for CD4 count to be analysed at the study laboratory. Children aged 18 months and older were tested for HIV, unless a result from a recent test was available. Those 0–18 months old were tested for HIV using DNA PCR methods, if their mothers were HIV positive. We report the total TB and HIV prevalence at that visit including both already diagnosed cases and patients diagnosed for the first time at the initial study visit.

All those who could provide a sputum sample had one taken for auramine smear and mycobacterial growth indicator tube (MGIT) culture - irrespective of the presence of symptoms suggestive of TB. Children who could not produce sputum were referred to the clinic for further investigation. TB and CD4 results were provided to the households at a visit about six weeks after the baseline visit. All index cases and household contacts who tested HIV positive were referred for either isoniazid preventative therapy (IPT) and/or antiretroviral therapy (ART). Those who had TB disease were referred for TB treatment and children under five were referred for assessment to their nearest clinic.

### Follow-up study visit

We now report the findings of a second study visit in 2010 made to the same households approximately one year after the first. All participants who were able to be re-contacted were individually asked whether they had been diagnosed with HIV or TB in the period between visits. If a positive response was received the date of diagnosis was elicited. Patient-held TB treatment cards were used to verify TB treatment occurring between visits. If a household member had died in the interim, we asked about the cause of death. All surviving participants had a standardised TB symptom screen performed at the second visit. Those with symptoms of TB had a sputum sample taken for smear and culture. Smears were examined under polarised light after auramine staining and sputum samples were cultured in liquid medium using the mycobacterial growth indicator tube system (Becton Dickenson Franklin Lakes, NJ). All baseline HIV seronegative individuals - including those reporting seroconversion between study visits - were offered rapid, on site HIV testing or laboratory ELISA HIV testing on blood or saliva specimens.

### Inclusion criteria

For the TB prevalence and incidence analyses, we only included household contacts who had a sputum result or were on TB treatment at their first study visit and who either died in the intervening year or were able to be contacted at the second visit. No index TB cases were included in the prevalence or prospective analysis. Incident TB was defined either as a new diagnosis of TB, neither present at the first household visit, nor diagnosed within 60 days following that first study visit. We also included as incident cases, contacts whose relatives reported that they died of TB in the intervening time. Those with prevalent TB at the first visit were considered to have an incident episode only if they were diagnosed with TB at least 8 months after the prior TB diagnosis.

Household contacts who tested negative for HIV at the first visit and had a follow up test at the second visit were included in the HIV incidence calculation. Incident HIV infections were defined as participants with a negative HIV rapid test at baseline and a positive HIV rapid test and/or laboratory HIV test one year later. Those who reported being tested HIV-infected between the first and final visits had a confirmatory rapid test at the second visit. HIV seroconversions were assumed to have happened at uniformly distributed times between first and second visits. Similar to the first study visit a year earlier, any participant at this second study visit who was diagnosed with HIV had an immediate CD4 count taken and then referred for either IPT or ART initiation depending on their CD4 count; those with positive auramine smear or mycobacterial culture were referred for initiation of TB treatment.

### Statistical methods

The sample size of this study was based on a TB prevalence survey [11] and was not designed to provide precise estimates of incidence rates or compare incidence rates between groups. For this reason, only descriptive statistics are reported. The binomial exact method was used to calculate 95% confidence intervals for the incidence and prevalence estimates.

## Results

Of 2,843 household contacts assessed at baseline, 2,337 (82%) were re-contacted at a median time of 360 days (IQR 341–404 days) after the first visit; 40 contacts died in the intervening period. The ratio of females to males at the baseline visit and approximately one year later was 1.37 and 1.44, respectively (p = 0.39). The median age of all participants at baseline was 18 years (IQR 8–34) and that of those re-assessed a year later was also 18 years (IQR 9–38). At both visits 42% of contacts were 0–14 years old. The 670 households of a TB index case had a median of 4 windows and 2 doors and the median household size was 5 people (IQR 3–7) per household.

### Baseline TB and HIV prevalence

Baseline total TB prevalence for the 2,377 household contacts who were revisited or had died in the meantime, was 9.2% (95% CI 8.0–10.5%). These cases were either found by active case finding or were already on TB treatment at the time of the first study visit. Similarly, total HIV prevalence at the first visit was 17.9% (95% CI 16.0–20.0%). TB prevalence among HIV-infected participants was 10.6% (95% CI 7.2–15.0%) and HIV prevalence among TB patients was 24.3% (95% CI 16.8–33.2%).

### Mortality

Of 40 household contacts who died in the intervening period between study visits, 11/23 (48%) were HIV-infected at baseline. Eight of the 40 (20%) reportedly died of TB or while on TB treatment; four of the eight tested for HIV at baseline and two of the four were HIV-infected.

### TB incidence

**Figure 1:**
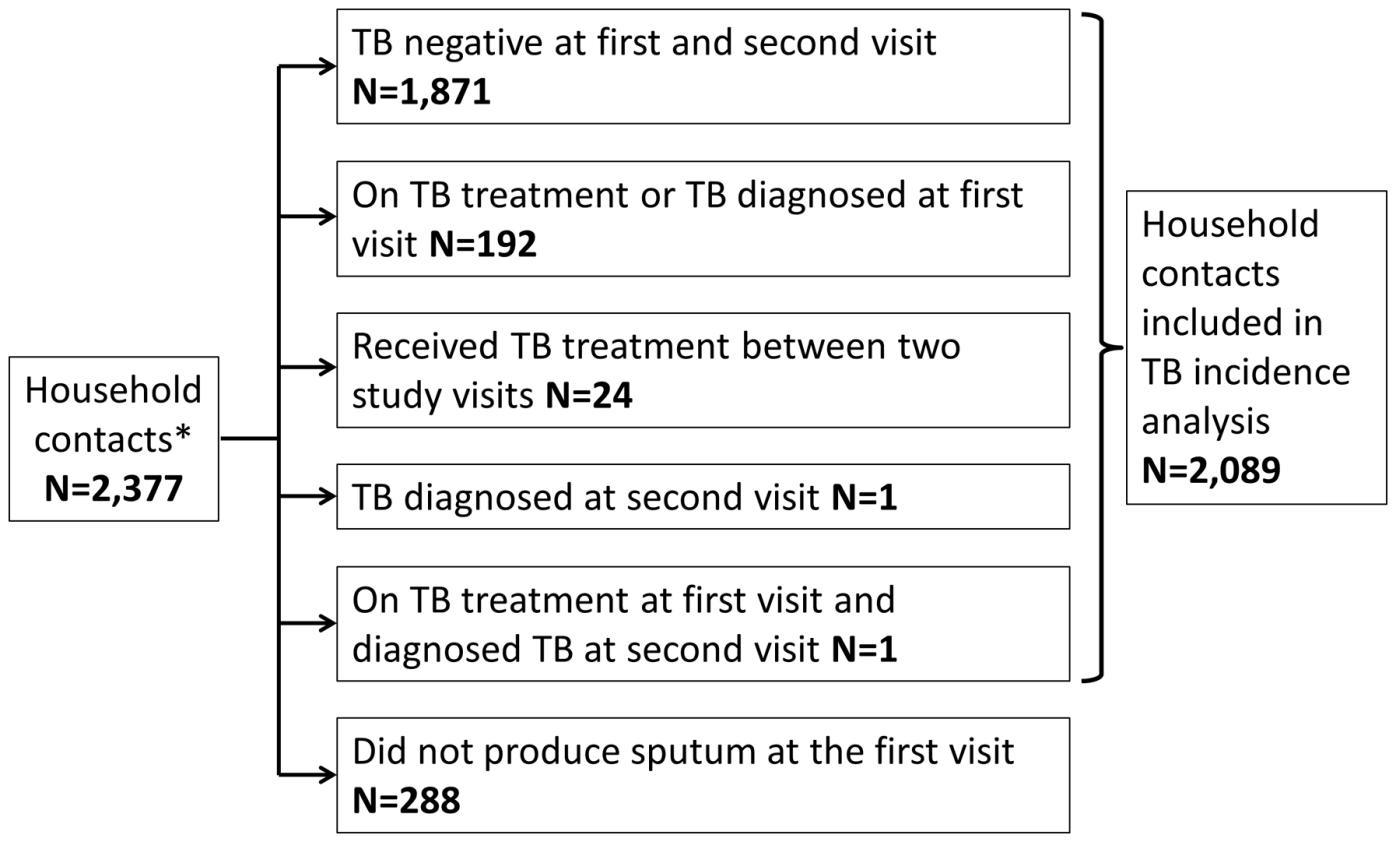
TB results of household contacts at the first household visit and at the second household visit. * Household contacts who were found again at follow-up visit or who died between visits were included in this study.

Overall, there were 26 incident TB cases in 2,089 people who had a documented culture result at baseline and who were followed up at the second study visit a year later (22), or died in the intervening period (4). Cumulative follow up time was 1,960 years during which 24 incident TB cases occurred in the time between the study visits, a median time of 170 days after the first study visit. A further two TB cases were diagnosed at the second household visit (Figure 1). The overall observed incidence rate of TB was therefore 1.3 per 100 person years (/100py) (95% CI 0.9–1.9/100py); that for individuals who were HIV-infected and HIV seronegative at baseline was 5.4/100py (95% CI 2.9–9.0/100py) and 0.7/100py (95% CI 0.3–1.4/100py), respectively. TB incidence rate for males and females was 1.4/100py (95% CI 0.7–2.5/100py) and 1.3/100py (95% CI 0.7–2.1/100py), respectively. The peak age group for TB was 35–54 year olds with an incidence rate of 2.4/100py (95% CI 1.0–4.7/100py). TB incidence rate for children 0–14 years of age and adults aged 15 and above were 1.2/100py (95% CI 0.5–2.3/100py) and 1.4/100py (95% CI 0.8–2.2/100py) respectively. A positive smear result in the index case appeared to show higher risk of incident TB (Table 1). Of 53 HIV infected household contacts who were referred for IPT at the baseline visit, 16 (27.1%) reported at the second visit that they received IPT. Two of those who were referred for IPT but did not take it, had an incident TB episode. None of 115 household contacts who reported that they received IPT since the first visit had an incident episode of TB.

**Table 1:** TB incidence and HIV incidence by individual and household-level sociodemographic characteristics of the cohort of household contacts of TB index cases in a high HIV prevalent and TB burden setting.

	Total (N = 2377)		TB Incidence (N = 2089)			HIV Incidence (N = 815)		
	N	%	N	per 100py	95% CI	N	per 100py	95% CI
**Total**	2377		26	1.3	0.9–1.9	18	2.2	1.3–3.4
**Individual-level characteristics**								
**Sex**								
Female	1403	59%	15	1.3	0.7–2.1	12	2.5	1.3–4.3
Male	974	41%	11	1.4	0.7–2.5	6	1.9	0.7–3.8
**Age category**								
0–14	1004	42%	8	1.2	0.5–2.3	2	0.7	0.1–2.6
15–34	716	30%	5	0.8	0.2–1.8	9	3.1	1.4–5.7
35–54	358	15%	8	2.4	1.0–4.7	4	3.7	1.0–9.2
55+	299	13%	5	1.8	0.6–4.2	3	2.1	0.4–6.0
**Education**								
none	706	30%	5	1.2	0.4–2.9	5	2.8	0.9–6.4
primary/secondary/tertiary	1660	70%	21	1.4	0.8–2.1	13	2.0	1.1–3.5
missing	11	0%	0			0		
**Employment**								
Student/minor	1273	54%	8	0.9	0.4–1.7	5	1.2	0.4–2.7
Employed (formal/informal)	202	9%	3	1.5	0.3–4.4	3	4.4	0.9–12.2
Unemployed/able	559	24%	9	1.7	0.8–3.3	8	4.2	1.8–8.0
Pensioner/unable to work	302	13%	5	1.8	0.6–4.2	2	1.5	0.2–5.2
missing	41	1%	1			0		
**Household-level characteristics**								
**Number of people in household**								
2–4	560	24%	8	1.7	0.7–3.4	7	3.9	1.6–7.8
5–7	952	40%	10	1.3	0.6–2.3	5	1.6	0.5–3.7
8+	865	36%	8	1.1	0.5–2.2	6	1.8	0.7–3.9
**Housetype**								
House/Other	1786	75%	16	1.1	0.6–1.7	13	2.1	1.1–4.2
Shack	580	24%	10	2.1	1.0–3.9	5	2.4	0.8–5.6
missing	11	0%	0					
**Average number of people per room**								
0–1	583	25%	7	1.4	0.6–2.9	5	2.4	0.8–5.5
1–2	1032	43%	9	1.2	0.5–2.1	8	2.4	1.0–4.7
>2	762	32%	10	1.3	0.8–2.9	5	1.8	0.6–4.1
**Signs of indoor smoking**								
No	2255	95%	24	1.3	0.8–1.9	17	2.2	1.3–1.4
Yes	117	5%	2	2.7	0.3–9.4	1	2.8	0.1–14.9
missing	5	0%	0			0		
**Index smear result**								
Negative	300	13%	5	2.0	0.6–4.5			
Scanty	107	5%	2	2.2	0.3–7.7			
Moderate	195	8%	4	2.5	0.7–6.3			
Numerous	291	12%	6	2.5	0.9–5.4			
Not available/not performed	1484	62%	9	0.7	0.3–1.4			

Household-level characteristics are features of the household or index relative to each contact.

When we included household contacts who did not have a sputum taken for auramine smear and MGIT culture at baseline in the incidence calculation - assuming them to be TB negative at baseline - the overall TB incidence rate was also 1.3/100py (95% CI 0.9–1.8/100py).

### HIV Incidence

**Figure 2:**
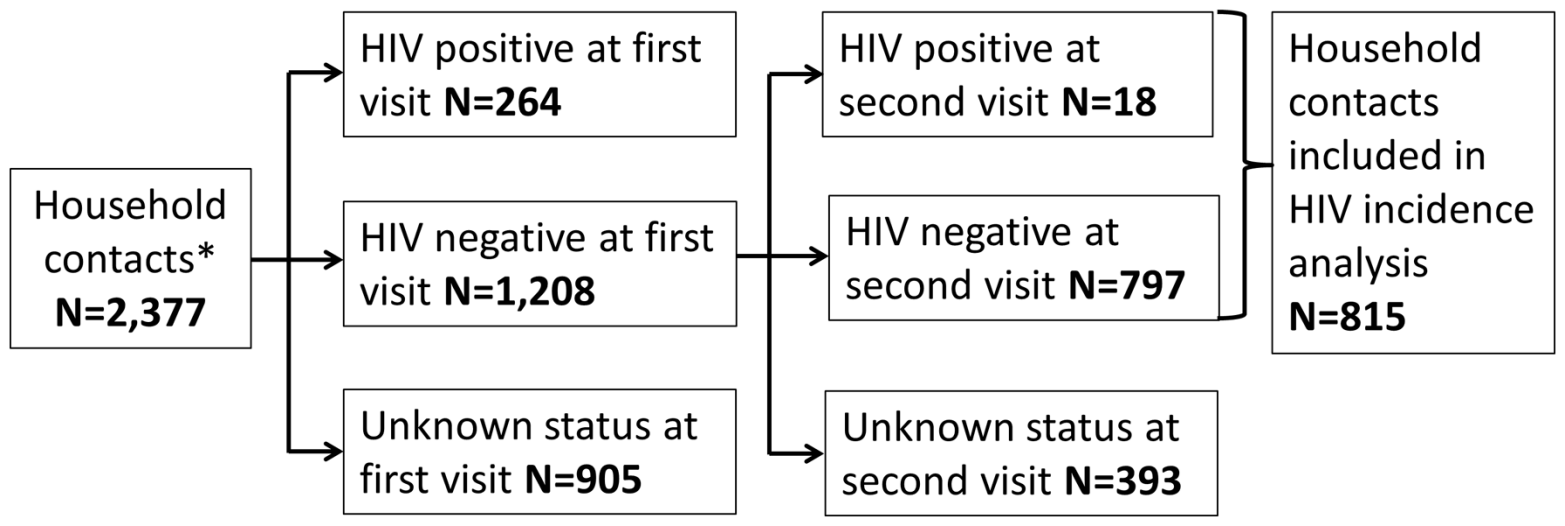
HIV results of household contacts at the first household visit and at the second household visit. * Household contacts who were found again at follow-up visit or who died between visits were included in this study.

Of the 1,472 household contacts HIV-tested at baseline, 1,208 were HIV seronegative. Of the HIV seronegative contacts, 815 (67%) were tested again at the second household visit (Figure 2). There were 18 seroconversions in this group and 823 person years of exposure time, reflecting an overall HIV incidence rate of 2.2/100py (95% CI 1.3–8.4/100py). Twelve females and six males had a documented seroconversion, reflecting an HIV incidence rate of 2.5/100py (95% CI 1.3–4.3/100py) and 1.8/100py (95% CI 0.7–3.8/100py), respectively. The peak incidence rate was in the age stratum 35–54 years at 3.7/100py (95% CI 1.0–9.2/100py). Higher HIV seroconversion rates occurred in households with fewer people per room (Table 1).

Of note, 265 of 905 (29%) participants who refused HIV testing at the baseline visit agreed to HIV testing at the second household visit - 20 (7.5%) were HIV-infected. These participants were not included in incidence calculations. Fourteen contacts were referred for ART at the first study visit – 4 reported that they had started ART.

## Discussion

In the year following an intensified case finding visit for TB and HIV, approximately 1.3/100py and 2.2/100py of household contacts of TB index cases developed TB, or were newly infected with HIV, respectively. Although subsequent cases of TB may have been reduced by the single intensified case finding strategy as has previously been suggested [12], the point estimate of annual TB incidence rate in contacts of index TB cases we report is still higher than the population level estimate of incidence for the entire district based on TB notifications in 2009. TB incidence rate among HIV-infected individuals we found in this study – 5.4/100py - is similar to the incidence rate reported in a clinical cohort of HIV-infected individuals not taking antiretrovirals in Soweto, about 120km from Matlosana - 4.7/100py (95% CI 4.0–5.8) [13].

The point estimate of HIV incidence in retested participants we report was also high when compared to the modelled HIV incidence estimate for North West Province in 2010 which was 0.79% for the general population [14]. This, despite participants receiving HIV counselling and testing one year previously - counselling and HIV testing has been shown to reduce sexual risk behaviour in discordant couples [15], [16]. Moreover, this health sub-district has a well-functioning antiretroviral program with, reportedly, over 30,000 individuals receiving HAART, and free-of charge condoms are widely available at all 16 primary care community clinics, making the high HIV incidence we report particularly concerning.

Although a relatively large number of individuals were followed up, the sample size was too small to identify statistically significant risk factors for either incident TB or HIV, illustrating the large sample sizes required for studies with short follow up - even in high HIV incidence and high TB burden settings - to identify risk factors for TB disease and acquiring HIV infection. A second limitation is that two different TB case finding strategies were used at the first and second household visits and additional incident cases might have been identified if all participants – irrespective of TB symptomatology - had been asked to provide a sputum specimen for smear and mycobacterial culture, as was done at the first study visit when only 11% of TB cases were symptomatic. A third limitation is that no genotyping was done to link transmissions between cases and household contacts. Fourthly, refusal rates for HIV testing were high at both visits, 39% at first visit and 32% at the second visit, which may have resulted in biased estimates of HIV prevalence and incidence. Finally, the environment has altered since our study. Examples of improvements are: better TB diagnostics are available, more people have been started on antiretroviral therapy, and more HIV-infected adults are receiving IPT [17]. If the study were to be repeated now, lower HIV and TB incidence may be recorded. We therefore recommend that studies such as this be repeated cyclically to demonstrate implementation effectiveness of HIV and TB related interventions.

The high incidence rates of both HIV and TB found in this study suggest urgent action is required to control TB transmission, to prevent TB disease and to prevent new HIV infections. We posit that the efficacy of contact tracing for TB control purposes might be improved by a second intensified case finding visit and by providing preventive treatment against TB for both HIV-infected and HIV-seronegative contacts; this would of course have major cost implications. Earlier diagnosis of HIV with initiation of antiretroviral therapy at diagnosis will assist in preventing new HIV infections and TB cases [18]; but methods of encouraging cyclical HIV retesting [19] and ensuring linkages to care must be rigorously assessed before widespread implementation. Finally, there is a need for similar studies of TB and HIV incidence both in contacts of TB cases and in other populations to better understand TB and HIV transmission dynamics and to infer effectiveness of interventions to curb both epidemics.
